# The Diagnostic Dilemma of Splenic Non-Hodgkin’s Lymphoma and Splenic Abscess: A Narrative Review

**DOI:** 10.7759/cureus.31944

**Published:** 2022-11-27

**Authors:** Zakir A Khan, Marium Aisha, Christopher S Farkouh, Tamara Tango, Leyla Bereka, Hoor Ul Ain, Naod F Belay, Matthew Farkouh, Qaisar Ali Khan

**Affiliations:** 1 Surgery, Khyber Teaching Hospital, Peshawar, PAK; 2 Medicine and Surgery, Civil Hospital Khairpur, Sukkur, PAK; 3 Dermatology, Rush Medical College, Chicago, USA; 4 Internal Medicine, Faculty of Medicine Universitas Indonesia, Depok, IDN; 5 Internal Medicine, Fatima Jinnah Medical College, Lahore, PAK; 6 Internal Medicine, Jinnah Medical College, Peshawar, PAK; 7 Geriatric Medicine, Michigan State University, East Lansing, USA; 8 Emergency Medicine, Ponce Health Sciences University, Ponce, USA; 9 Internal Medicine, Divisional Headquarter Hospital (DHQ) Kohat Development Authority (KDA) Kohat Teaching Hospital, Kohat, PAK

**Keywords:** lymphadenopathy, splenic abscess, extra-nodal, total splenectomy, primary non-hodgkin’s lymphoma

## Abstract

Non-Hodgkin's lymphoma (NHL) is a lymphoproliferative disorder that principally displays lymph node involvement but can also spread to extranodal sites such as the spleen. Primary splenic NHL arises in the spleen and, due to its atypical presentation, can sometimes present similarly to other splenic conditions. This review aims to highlight how primary splenic NHL can be effectively differentiated from other splenic conditions, such as splenic abscesses. PubMed, MEDLINE, Scopus, Google, and Google Scholar were used to identify articles mainly focused on splenic non-Hodgkin's lymphoma and splenic abscess. The search was limited to articles published from January 2005 to November 2022. Of the 229 total articles amassed, only 34 were selected and narratively reviewed. From a thorough review of the current literature, it is evident that splenic NHL displays a similar clinical picture to other splenic conditions, namely splenic abscesses. One cannot easily differentiate between the two conditions, both clinically and via diagnostic imaging. Lymphadenopathy, a hallmark sign of nodal NHL, may or may not be present in splenic NHL. Ultimately, splenectomy with biopsy and immunohistochemical staining (IHC) may be required to confirm the diagnosis. In cases of suspected splenic NHL or splenic abscess with little-to-no symptomatic improvement after medication administration, splenectomy followed by histopathological examination may be required for a definitive diagnosis and proper treatment.

## Introduction and background

Non-Hodgkin's lymphomas (NHL) are a heterogeneous group of lymphoproliferative malignancies that are less predictable than Hodgkin's Lymphomas and have a far greater predilection for extranodal location dissemination. NHL is one of the most common hematological malignancies worldwide, characterized by an infiltration of small to medium-sized lymphocytes rich with cytoplasm and irregular nuclei, plasma cells, and monocytoid B-cells [1]. The incidence of NHL in the United States (US) was 18.6/100,000 in 2017, 1.68 times higher than in 1975 [2]. Splenic NHL may result from the extension of primary nodal NHL or arise de novo in the spleen, the latter of which we refer to as splenic NHL. Splenic NHL presents similarly to a splenic abscess based on both clinical and radiological evaluations. This review mainly highlights the techniques by which a clinician can differentiate between these two conditions.

## Review

**Figure 1:**
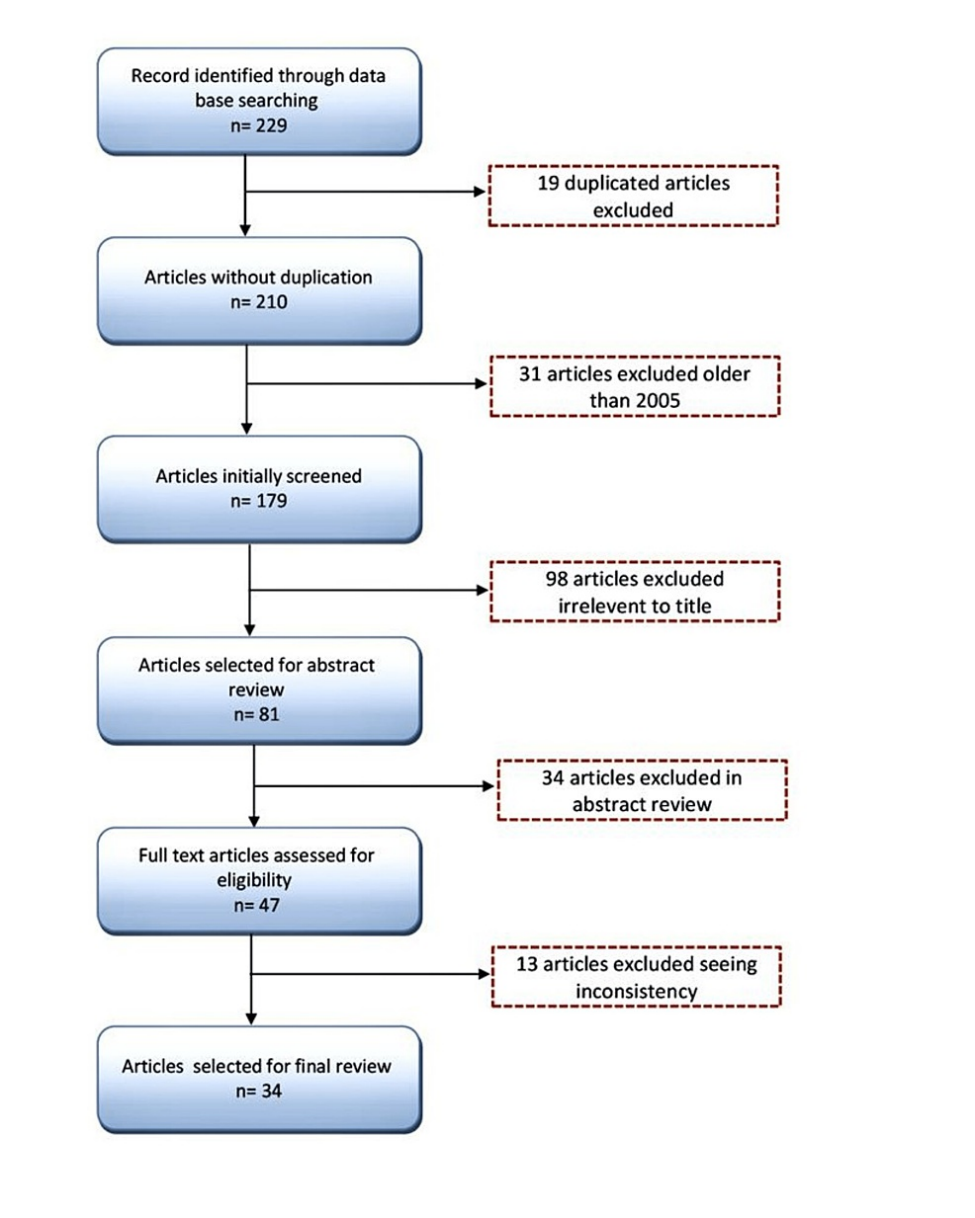
Flow chart of literature search.

A literature search was performed using PubMed, MEDLINE, Scopus, Google, and Google Scholar. Our search was focused on splenic non-Hodgkin’s lymphoma and splenic abscess. Search terms included "splenic non-Hodgkin’s lymphoma" and "splenic abscess." All authors reviewed the titles of the articles found by the initial search. Titles suggestive of splenic NHL or splenic abscess were selected for abstract review. Articles not focused on the clinical manifestations, laboratory evaluation, diagnosis, treatment, and prognosis of splenic NHL and splenic abscess were excluded. A total of 229 articles met the search criteria applied in Figure 1. Of these, 19 duplicated articles were excluded. Out of the 210 unduplicated articles, 31 were excluded because they were published before 2005. Ninety-eight titles appeared to be irrelevant to splenic abscess and splenic NHL and were thus excluded. Thirty-four articles were excluded from the abstract review. Lastly, 13 articles were excluded due to inconsistency. The remaining 34 articles successfully fit the inclusion criteria and were subsequently reviewed.

Splenic NHL and splenic abscess

NHL primarily involves lymph nodes, with their enlargement generally being the first sign of disease leading to a diagnosis [3]. In some cases, NHL may involve extranodal lymph nodes, including the spleen, brain, nose, paranasal sinuses, lungs, intestines, liver, and testes [4]. Splenic abscesses are recognized to be complications of infective endocarditis, immunodeficiency, neoplastic changes, physical trauma, metastatic infection, or diabetes with often isolated pathogens such as streptococcus, staphylococcus, and *Klebsiella pneumonia*. [5] NHL, conversely, arises due to chromosomal translocation or mutation/deletion. NHL may be associated with various factors, including but not limited to, inherited immune disorders, infections, environmental factors, immunodeficiency states, and chronic inflammation. Pathogens attributed to the different types of NHL include Epstein-Barr virus, human T-cell lymphotropic virus type 1 (HTLV-1), hepatitis C virus (HCV), and *Helicobacter pylori* infection [5,6].

Clinical manifestations

Splenic NHL and abscess both present with B symptoms, including fever, sweating, weight loss, and left upper quadrant pain. Weight loss is more prominent in splenic NHL and chronic splenic abscesses, whereas weight loss is rare in acute abscesses [3,7,8]. Lymphadenopathy, a classical sign of NHL, can provide physicians with a clearer understanding of the spread of NHL in patients. Importantly, however, the spread of NHL to the abdominal organs is not necessarily accompanied by peripheral lymphadenopathy, leaving the physician to explore other means of understanding the patient's degree of malignancy. Enlarged abdominal lymph nodes are nonspecific and can be present in both conditions but are not necessary for diagnosis; hence, one can suspect either of these conditions without the presence of enlarged lymph nodes [9]. Splenomegaly is commonly present in both conditions. The progression of splenomegaly development and splenic size can provide clues in distinguishing splenic NHL or abscess. The rapid development of splenomegaly and a splenic size of more than 13 cm mostly favor the diagnosis of splenic NHL compared to a splenic abscess [10].

Laboratory evaluation

Both the conditions of splenic NHL and splenic abscess can cause leukocytosis, anemia, an increased ESR, and an elevated lactate dehydrogenase (LDH) [11,12]. These similar initial laboratory findings make it challenging to distinguish the specific underlying cause; however, anemia in the case of splenic NHL tends to be of the hemolytic type or anemia of chronic disease. In addition, thrombocytosis, lymphocytosis, and pancytopenia are more predictive of splenic NHL. Other diagnostic biological markers for NHL include serological, immunophenotypic, and molecular markers [9,11,12]. Immunoreactivity, i.e., the presence of Bcl-2, which is an antiapoptotic protein, can distinguish the malignant cells of lymphoma from benign reactive B cells in the marginal zone of the spleen [11,12]. In contrast to NHL, serum protein and immunoglobulin tests are not helpful in splenic abscesses; however, blood cultures may be optimistic [12]. Thus, further workup is needed to evaluate the exact cause.

Radiological assessment

A plain radiograph is not appropriate in the evaluation of a splenic abscess as it yields low sensitivity. Indirect signs such as pleural effusions with or without the presence of left basal atelectasis or that of an elevated left hemidiaphragm may indicate splenic abscesses where NHL presents as splenomegaly greater than 13 cm [10]. On CT scans/ultrasound, a splenic abscess is typically poorly demarcated, ranging from a hypoechoic to a hyperechoic appearance coupled with ascites and an adjacent pleural effusion. NHL manifests as small, hypoechogenic, circumscribed nodules on ultrasound [13]. A hypodense lesion adorned with smooth borders and a necrotic center is commonly observed in splenic NHL, often warranting splenectomy to confirm the diagnosis [7]. Radiological assessment is important but can be non-conclusive, especially if tumor necrosis is present.

Biopsy and immunohistochemical staining

As the symptoms and supporting investigations of splenic NHL may be similar to those seen in splenic abscesses, it can be challenging for a physician to differentiate between the two conditions [14]. Splenic NHL can mask the splenic abscess and lead to misdiagnosis of the pathology [3,7,15-17]. Therefore, it is crucial to understand the subtypes of splenic NHL, their associated infections, and their management. Generally, the subtypes of splenic NHL consist of diffuse large B cell lymphoma, splenic marginal zone lymphoma (SMZL), splenic diffuse red pulp lymphoma, and the hairy cell leukemia variant HCL-v [18]. Biopsy with immunohistochemical staining (IHC) can be used to distinguish NHL subtypes. Different types of biopsy, such as core biopsy or fine needle aspiration cytology (FNAC), can hit the diagnosis, but as splenectomy is both therapeutic as well as diagnostic, an excisional biopsy could be the gold standard. IHC can show different cell markers and help in distinguishing the types of splenic lymphoma. Other advanced modalities may also be used, such as cytogenetic studies, polymerase chain reaction (PCR), and fluorescent in situ hybridization (FISH) [19]. Of note, among the NHL subtypes, SMZL has a loose association with chronic hepatitis C infection [19]. Treating the infection with antiviral drugs can often cause these lymphomas to undergo remission. A splenectomy can at times lead to remission if the use of antiviral drugs is unable to yield desirable outcomes or if the patients do not have a history of chronic hepatitis C infection [20].

Classical treatment and recent advancements

There are several treatments of choice to manage splenic NHL, the selection of which depends highly on the stage and type of lymphoma. Conventionally, an early splenectomy is chosen due to its ability to provide a therapeutic outcome. With the ever-growing archive of case reports and emerging literature, physicians have been able to better differentiate NHL from splenic abscesses. In recent literature, physicians were able to perform splenectomies followed by histopathological examinations to make accurate diagnoses as the patients did not respond to the antibiotic’s treatment [7,15-17]. In addition, in cases where transformations are suspected, such as those seen in nodular lesions with augmented fluorodeoxyglucose uptake, there are clear indications that splenectomy is necessary [18]. In patients with multiple concomitant comorbidities and/or advanced age, surgeons should be cautious in recommending splenectomy, as a rupture is a concern. Patients may remain treatment-free after the surgery [21], although there still exists the possibility for short-term and long-term complications such as perioperative intolerance, immune suppression, and chronic infections [21,22]. Other treatment options for NHL include radiation, chemotherapy, and the monoclonal antibody rituximab [23-25]. A combination of rituximab with chemotherapy to treat an aggressive tumor such as diffuse large B cell lymphoma of the spleen is proven to have a more favorable outcome, as indicated by the increased survival of patients afflicted with NHL. A study reported the overall response rates were 88% with rituximab, 83% with rituximab plus chemotherapy, and 55% with chemotherapy alone, while the three-year survival rates were 95%, 100%, and 55%, respectively [25]. Current guidelines recommend the treatment of SMZL be dependent upon the presence or absence of splenomegaly, cytopenia, and/or progressing nodal illness. Upon the resolution of splenomegaly, normalized blood cell counts, and a negative blood flow cytometry, a complete response (CR) is obtained to evaluate the patient [26,27].

The gold standard treatment for a splenic abscess is splenectomy, although other forms of management can be utilized in a case-dependent manner. Percutaneous aspiration serves as a possible second line of treatment as it involves a less invasive course suitable for those at high risk for surgery, avoiding the risk of a fulminant infection. Although it may serve as a preferred second-line treatment, percutaneous drainage is not as effective in patients with a past history of multilocular, poorly demarcated, necrotic abscesses, hence making splenectomy the superior treatment [28,29]. Although high-dose parenteral broad-spectrum antibiotics are to be administered while diagnostic tests are pending, medical treatment alone is not recommended, as mortality rates depict a 50% mortality rate in patients only managed with antibiotics [28].

Prognosis

The prognosis of NHL patients can be assessed using a prognostic score. The Italian Intergroup for Lymphoma (ILL) previously proposed the use of a prognosis score, with their study showing a five-year cause-specific survival rate (CSS) of NHL at 76% [30]. Worse outcomes (shorter CSS) were associated with lower hemoglobin values (Hb < 12 g/dL), and lower albumin levels (albumin level <3.5 g/dL, elevated LDH) [30]. Another prognostic score known as the hemoglobin-platelet-LDH-extra-hilar-lymphadenopathy (HPLL) score developed by the SMZL Study Group demonstrated a more concise and accurate stratification power [31]. In general, the prognosis of NHL depends on the stage of the NHL with most cases having a 10-year median survival [32]. Splenic abscess prognosis is multifactorial - there is no single risk factor that can predict one’s severity of disease accurately. The APACHE II severity scoring system could be a useful tool in comparing the mortality and survival rates, which show statistically significant higher scores in the mortality group [33]. Although splenectomy might lead to a more favorable outcome, the ultimate prognosis rests on the underlying pathology predisposing the patient to the infection in the first place [34].

## Conclusions

It has been concluded that both primary splenic NHL and splenic abscess present clinically and radiologically in a similar manner. Without further investigation, no specific sign or symptom can predict the correct diagnosis. A splenic biopsy and immunohistochemical staining are necessary for the correct diagnosis. The proper approach to differentiating the two pathologies should take into account the patient’s pertinent past medical history and previous responses to medication. In cases of suspected splenic NHL or splenic abscess with little-to-no symptomatic improvement after medication administration, splenectomy followed by histopathological examination may be required for a definitive diagnosis and proper treatment.
